# Design of a Chimeric Multi-Epitope Vaccine (CMEV) against Both *Leishmania martiniquensis* and *Leishmania orientalis* Parasites Using Immunoinformatic Approaches

**DOI:** 10.3390/biology11101460

**Published:** 2022-10-05

**Authors:** Kentaro Imaizumi, Thararat Phurahong, Suradej Siripattanapipong, Kiattawee Choowongkomon, Saovanee Leelayoova, Mathirut Mungthin, Teerasak E-kobon, Sasimanas Unajak

**Affiliations:** 1 Department of Biochemistry, Faculty of Science, Kasetsart University, 50 Ngam Wong Wan, Chatuchak, Bangkok 10900, Thailand; 2 Kasetsart Vaccine and Bio-Product Innovation Centre, Kasetsart University, 50 Ngam Wong Wan, Chatuchak, Bangkok 10900, Thailand; 3 Department of Microbiology, Faculty of Science, Mahidol University, Bangkok 10400, Thailand; 4 Department of Parasitology, Phramongkutklao College of Medicine, Bangkok 10400, Thailand; 5 Department of Genetics, Faculty of Science, Kasetsart University, 50 Ngam Wong Wan, Chatuchak, Bangkok 10900, Thailand

**Keywords:** leishmaniasis, chimeric multi-epitope vaccine, immuno-informatics, in silico vaccine design, reverse vaccinology, neglected tropical diseases

## Abstract

**Simple Summary:**

Leishmaniasis, caused by parasites from the genus *Leishmania*, is one of the neglected tropical diseases that is particularly problematic in tropical regions. Vaccine development is anticipated, but the diversity of pathogens poses a challenge. In this study, we designed a vaccine that is expected to be effective against both *Leishmania martiniquensis* and *Leishmania orientalis*, two different species isolated in Thailand. Predicted antigenic proteins and their epitopes were extracted from the draft genomes of these two species, and a chimeric multi-epitope vaccine was constructed in silico. The immunogenicity, chemical, and structural properties of the designed protein molecules suggest that this molecule could be properly synthesized in a heterologous expression system and induce responses in the inoculated host immune system. Based on these results, further experiments are required to determine the practical application of this vaccine candidate.

**Abstract:**

Leishmaniasis is a parasitic disease caused by protozoan flagellates of the genus *Leishmania*. Recently, *Leishmania martiniquensis* and *Leishmania orientalis*, emerging species of *Leishmania*, were isolated from patients in Thailand. Development of the vaccine is demanded; however, genetic differences between the two species make it difficult to design a vaccine that is effective for both species. In this study, we applied immuno-informatic approaches to design a chimeric multi-epitope vaccine (CMEV) against both *L. martiniquensis* and *L. orientalis*. We identified seven helper T lymphocyte (HTL) epitopes, sixteen cytotoxic T lymphocyte (CTL) epitopes, and eleven B-cell epitopes from sixteen conserved antigenic proteins found in both species. All these epitopes were joined together, and to further enhance immunogenicity, protein and peptides adjuvant were also added at the N-terminal of the molecule by using specific linkers. The candidate CMEV was subsequently analyzed from the perspectives of the antigenicity, allergenicity, and physiochemical properties. The interaction of the designed multi-epitope vaccine and immune receptor (TLR4) of the host were evaluated based on molecular dockings of the predicted 3D structures. Finally, in silico cloning was performed to construct the expression vaccine vector. Docking analysis showed that the vaccine/TLR4 complex took a stable form. Based on the predicted immunogenicity, physicochemical, and structural properties in silico, the vaccine candidate was expected to be appropriately expressed in bacterial expression systems and show the potential to induce a host immune response. This study proposes the experimental validation of the efficacy of the candidate vaccine construct against the two *Leishmania*.

## 1. Introduction

Leishmaniasis is a parasitic infectious disease caused by more than twenty species of the protozoan flagellates of the genus *Leishmania,* which are transmitted with the phlebotomine sandfly as the vector [1]. *Leishmania* causes three major clinical forms of infection, including cutaneous leishmaniasis (CL), mucocutaneous leishmaniasis (MCL), and visceral leishmaniasis (VL), which is a fetal and systemic disease if left untreated [2]. In Thailand, two recent emerging species, *L. martiniquensis* and *L. orientalis* (formerly *L*. *siamensis*), are spreading and causing leishmaniasis [3,4,5,6,7]. Anti-leishmanial drugs such as amphotericin B and pentavalent antimonials are effective against this disease, but the chemotherapeutic drugs have limitations, which are high cost, adverse effects, availability, and drug resistance among the pathogens [8,9]. Therefore, vaccine development is needed as an alternative against *Leishmania* parasites.

Vaccines based on live-attenuated *Leishmania* parasites, crude extracts of *Leishmania* parasites, recombinant proteins and synthetic peptides, and DNA vaccines have been studied [10]. Vaccines that rely on the whole parasite itself are not practical due to the concerns of safety and the difficulty in culturing the parasites. Therefore, it is necessary to develop subunit recombinant vaccines that target the pathogen instead of using killed or attenuated whole cell vaccines. The *Leishmania* homologue for receptors of activated C kinase (LACK) [11], glycoprotein leishmaniolysin (gp63) [12], and promastigote surface antigen 2 (PSA 2) are reported as potential antigens [13]. Vaccination using polyprotein antigens (Leish-110f and LEISH-F3) reduced *Leishmania* infection in mouse models [14,15]. However, none of these experimental vaccine candidates have progressed in human trials but mainly in murine and canine [16,17]. Moreover, studies of vaccines against recently emerging *Leishmania* parasites, *L. martiniquensis* and *L. orientalis*, are limited.

Multi-epitope vaccines targeting conserved or common antigens are desirable for vaccines against parasitic diseases caused by multiple species of *Leishmania*. Immuno-informatic approaches were used in the designing of multi-epitope vaccines against pathogens such as human immunodeficiency virus (HIV), *Vibrio cholerae*, and *Staphylococcus aureus* [18,19,20]. In *Leishmania*, the design of multi-epitope vaccines targeting a single species was reported [21,22,23]. A higher induction of the immune response and protection was observed in antigens with multiple epitopes compared to mixed recombinant proteins [24]. Similar approaches may be used to develop vaccines effective against diverse strains or among closely related species.

In the current study, we designed a multi-epitope vaccine against both *L. martiniquensis* and *L. orientalis* using immuno-bioinformatic tools. The B-cell and T-cell epitopes that are conserved between two species of *Leishmania* parasites were explored, and the most promising combination was predicted based on the antigenicity, physicochemical and structural properties, and interaction with immune receptors. To our best knowledge, this is the first report of a blueprint for a candidate vaccine against the two emerging parasites in Thailand.

## 2. Materials and Methods

### 2.1. Screening of Antigenic Proteins

The proteome data for *L. orientalis* and *L. martiniquensis* were predicted from the published genomes of *L. orientalis* isolate PCM2 and *L. martiniquensis* isolate PCM3 [25] using the Augustus program (http://augustus.gobics.de/ (accessed on 3 February 2022)). The protein sequences were submitted to the antigenic prediction program available at the database of protozoan virulence proteins (ProtVirDB) (http://bioinfo.icgeb.res.in/protvirdb/home.html (accessed on 5 February 2022)). This program provided the number of antigenic regions (hit count) associated with the prediction scores of 0 to 1 for the possibility of an antigenic region within the input protein sequences. The output from the prediction program was processed to summarize the result using R scripts. The proteins with the highest number of antigenic regions were selected for the following analysis.

### 2.2. Selection of Common Antigenic Proteins between the Two Species of Leishmania

**Figure 1:**
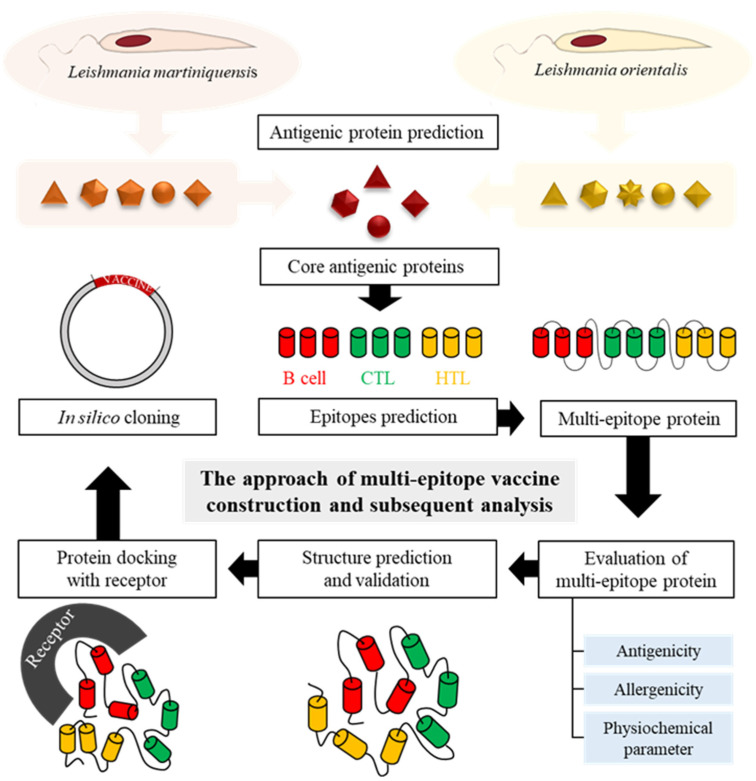
Schematic representation of the multi-epitope vaccine construction and subsequent analysis of this study.

After antigenic proteins were collected, the protein sequences were subsequently aligned using Clustal Omega. Common antigenic proteins were found among *L. martiniquensis* and *L. orientalis* based on the similarity of protein sequences. The selected proteins were used for further analysis, which is represented in Figure 1.

### 2.3. Prediction of B-Cell Epitopes

Common antigenic proteins from both *Leishmania* species were submitted to the ABCPred server (http://crdd.osdd.net/raghava/abcpred/ (accessed on 22 February 2022)), with application of the standard threshold (0.51), 10-mer length epitopes were selected to predict the antigenicity [26].

### 2.4. Prediction of Cytotoxic T-Lymphocyte (CTL) Epitopes

CTLs, also known as CD8^+^ T cells, play a major role in controlling bacteria, virus, and parasite infection through their cytotoxic activity. CTLs epitopes were predicted using NetCTL 1.2 server (https://services.healthtech.dtu.dk/service.php?NetCTL-1.2 (accessed on 22 February 2022)), with the standard threshold (0.75), and all parameters were kept at default [27]. Epitopes were analyzed for allelic supertypes A2, A3, and B7, which provide maximal population coverage [28].

### 2.5. Identification of Helper T-Lymphocyte (HTL) Epitopes

HTLs play an important role in the induction of both humoral and cellular immune responses. HTLs epitopes were identified by using IEDB (http://tools.iedb.org/mhcii/ (accessed on 22 February 2022)) with the SMM-align (NetMHCII 2.3) method, and specified whether binding to 15 alleles of human HLA-DR [29]. The epitopes were ranked based on their affinity to bind their receptor, which was evaluated by IC50 and percentile rank. The lower percentile rank and IC50 value, corresponding to high affinity, were chosen.

### 2.6. Prediction of Antigenicity of Epitopes

The antigenicity of the selected epitopes was predicted by using VaxiJen v. 2.0 server (http://www.ddg-pharmfac.net/vaxijen/VaxiJen/VaxiJen.html (accessed on 22 February 2022)) with the server threshold of 0.5 for parasite model [30]. Only the epitopes with an antigenicity score more than 0.5 were selected to construct the multi-epitope vaccine.

### 2.7. Construction of Candidate Multi-Epitope Vaccine

To construct the multi-epitope vaccine, the selected epitopes were joined together by using suitable linkers and adjuvants. From the previous study [31], linkers GGGS, AAY, and KK were used to fuse the different components. To improve the immunogenicity, the 50S ribosomal protein L7/L12 (Locus RL7_MYCTU) (Accession no. P9WHE3) was chosen as a molecular adjuvant at the N-terminal region of the multi-epitope protein. Then, the pan HLA DR-binding epitope (PADRE: AKFVAAWTLKAAA) was added with the GGGS linker, followed by HTLs, CTLs, and the B-cell epitope with specific linkers (Figure 1). Finally, 6× His tag was added at the C-terminal region of CMEV for purification assays.

### 2.8. Antigenicity and Allergenicity

Antigenicity is the ability of an antigen to provoke an immune response against an infection. To evaluate the antigenicity of the vaccine construct, the VaxiJen v.2.0 server was used once more with the threshold for the parasite model (0.5). Then, the CMEV was subjected to an AllerTop v.2.0 server (https://www.ddg-pharmfac.net/AllerTOP/ (accessed on 25 February 2022)) [32]. AllerTop is a method based on the auto cross covariance (ACC) transformation of protein sequences into uniform equal-length vectors, classified based on a training set containing 2427 known allergens from different species and 2427 non-allergens. To evaluate the similarity with human proteins to reduce autoimmunity possibilities, a BLASTP was performed against the UniProtKB Human database.

### 2.9. Analysis of Physicochemical Properties

To evaluate the physicochemical properties, including 1. amino acid composition, 2. molecular weight, 3. theoretical pI, 4. aliphatic index, 5. estimated half-life for three model organisms (*Escherichia coli*, yeast, and mammalian cells), 6. instability index, and 7. The grand average of hydropathicity (GRAVY), ProtParam online server (https://web.expasy.org/protparam/ (accessed on 25 February 2022)) was used [33]. The solubility was assessed for the vaccine construct using the SOLpro server (http://scratch.proteomics.ics.uci.edu/ (accessed on 25 February 2022)), which predicts the propensity of a protein to be soluble upon overexpression in *E. coli* [34].

### 2.10. Simulation of Immune Response and Efficacy of the Candidate Vaccine

To further characterize the immune response of the multi-epitope vaccine, in silico immune simulations were conducted by the C-ImmSim server (https://kraken.iac.rm.cnr.it/C-IMMSIM/ (accessed on 23 March 2022)) [35]. C-ImmSim is an agent-based model that uses a position-specific scoring matrix (PSSM) for the prediction of immune interactions. Considering the immunization schedule for the *Leishmania* vaccine tested in BALB/c mice, chimeraT [36], three doses at an interval of 14 days were adopted. Therefore, the vaccine protein was administered 14 days apart at 1, 42, and 84 time-steps (each time-step is equivalent to 8 h in real-life and time-step 1 is injection at time = 0) with a total of 1050 simulation steps, and the remaining parameters were kept as the default.

### 2.11. Structure Evaluation, Modeling, and Validation

The secondary structures of the multi-epitope vaccine construct were predicted using the PSIPred online tool (http://bioinf.cs.ucl.ac.uk/psipred/ (accessed on 25 February 2022)) [37]. The tertiary structure of the final vaccine was predicted by AlphaFold2 using MMseqs2 [38]. The secondary structure composition was evaluated using 2struc (https://2struc.cryst.bbk.ac.uk/twostruc (accessed on 25 February 2022)) [39]. The generated model structure was submitted to the GalaxyRefine server (https://galaxy.seoklab.org/cgi-bin/ (accessed on 25 February 2022)), which performs repeated structure perturbation and subsequent overall structural relaxation [40]. The best model was selected and subjected to the Structure Validation Server (SAVESv6.0) (https://saves.mbi.ucla.edu/ (accessed on 26 February 2022)) selecting PROCHECK to generate a Ramachandran plot [41].

### 2.12. Prediction of B-Cell Epitope

Discontinuous or conformational B-cell epitopes, which are formed by protein folding that can bring the residues to form it, were found in >90% of B-cell epitopes. The final refined multi-epitope 3D structure (pdb file) was submitted to the ElliPro server with a threshold score of >0.5 to evaluate the presence of these epitopes [42].

### 2.13. Molecular Docking of Multi-Epitope Vaccine with TLR4

To evaluate the interaction between CMEV and the immune receptor, the ClusPro 2.0 server (https://cluspro.org (accessed on 5 July 2022)) was run to analyze their interaction affinity [43]. The receptor used in this study is TLR4 (PDB ID: 2Z63), which can directly be activated by some *Leishmania* molecules and induces Th1 stimulatory responses [44]. The PDB file of TLR4 and the vaccine were submitted to generate the data about the vaccine-receptor complex. To visualize hydrogen bonds and hydrophobic interactions between the ligand and receptor, the software LIGPLOT v.2.2 was applied [45]. The ClusPro 2.0 server and LIGPLOT v2.2 software were run to simulate and analyze possible interactions between the TLR4 (receptor) and ligand (CMEV), which is determined through the number of docked structures, the energy of the cluster center, and the lowest energy structure in the cluster based on the model score for the balanced coefficient set.

### 2.14. In Silico Cloning

To construct the plasmid harboring the multi-epitope sequence, codon optimization was performed using the Java Codon Adaptation Tool (JCat) server, which provides a codon optimized DNA sequence based on the selected organism [46]. In this study, the *E. coli* (strain K12) was chosen. The result includes optimized sequences and two more parameters, which are the codon adaptation index (CAI) and the percentage of GC content. For CAI, the ideal value is 1.0 and the GC content should be 30 to 70%. Finally, in silico cloning of the final construct in the expression vector, pET28a(+), was performed by SnapGene 6.0.2.

## 3. Results

### 3.1. Common Antigenic Proteins among L. orientalis Isolate PCM2 and L. martiniquensis Isolate PCM3

From 8990 and 9577 proteins derived from the genomes of *L. orientalis* isolate PCM2 and *L. martiniquensis* isolate PCM3, the prediction program yielded 21 and 30 putative predicted antigenic proteins that contained more than 200 antigenic regions. The k59_9180.g3154 protein of *L. orientalis* (total hit score = 370) and CN030410.1.g884 protein of *L. martiniquensis* (total hit score = 335) had the highest summation of the hit scores.

**Figure 2:**
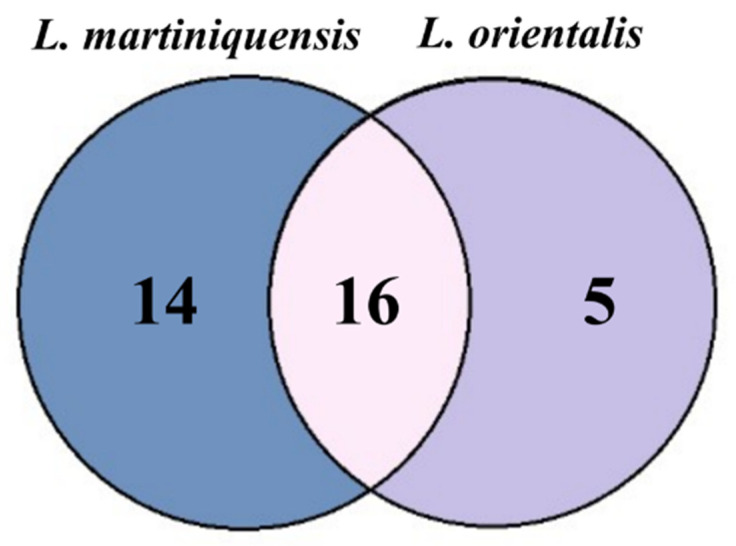
Venn diagram of common antigenic proteins between two species of parasites, *L. martiniquensis* isolate PCM2 and *L. orientalis* isolate PCM3.

The alignment of the multiple sequences aids to identify the common proteins between *L. martiniquensis* and *L. orientalis*. Sixteen proteins were observed as a common protein; five proteins from *L. orientalis* and fourteen proteins from *L. martiniquensis* were classified as unique proteins (Figure 2). The identities between proteins were determined using pairwise alignment. The result found that CN030429.1.g7028 and k59_748.g6249 had the highest identity while CN030420.1.g3164 and k59_328.g2467 had the lowest identity. Then, the core proteins were used for further analysis (Table 1).

### 3.2. Prediction of B-Cell Epitopes

The ABCpred server predicted linear B-cell epitopes for all core proteins. The common epitopes from each core protein that were found in both *L. martiniquensis* and *L. orientalis* with the highest score (>0.75) were selected. The antigenicity of epitopes was evaluated by VaxiJen 2.0 using threshold >0.5 for the parasite model. Eleven B-cell epitopes, which had a score higher than the threshold cut-off, were collected for further analysis (Supplementary Table S1).

### 3.3. Identification of CTL Epitopes and the Immunogenicity

We used the NetCTL 1.2 server to identify CTL receptor-specific immunogenic epitopes for all core proteins. For the CTL epitopes, allelic supertypes A2, A3, and B7 were predicted; only epitopes with a score of 0.75 or higher were selected, as these epitopes are the most likely to be immunogenic for CTLs. Furthermore, the immunogenicity of the predicted CTL epitopes was analyzed by the IEDB immunogenicity prediction module. Only epitopes with positive and the highest immunogenicity scores were considered for vaccine design; a total of 16 CTLs with scores above 0.75 were used for vaccine construction (Supplementary Table S2).

### 3.4. Identification of HTL Epitopes

We used the IEDB MHC-II epitope analysis tool to generate epitopes. The low percentile ranking and low IC50 values represent the high immunogenicity of the epitopes (Supplementary Table S3).

### 3.5. Construction of Multi-Epitope Vaccine

**Figure 3:**
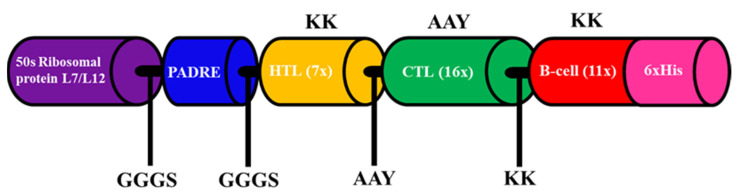
Schematic representation of the multi-epitope vaccine construct. Adjuvants and epitopes were connected by the linkers shown in the figure.

The best 34 epitopes that had an antigenic score higher than 0.5 (for the parasite model) were collected as a component of the final vaccine (Table 2). To construct a multi-epitope vaccine, suitable adjuvants were chosen, which were 50S ribosomal protein L7/L12 and PADRE peptide [47,48]. Both adjuvants were connected by the GGGS linker at the N-terminal region. Then, the GGGS linker was inserted once to join 7× HTL epitopes and the adjuvant region, while HTL epitopes were connected by the KK linker. A total of sixteen CTL epitopes were attached using AAY linkers and connected to the previous region with the KK linker. Between the last CTL epitope and first B-cell epitope, the AAY linker was added, and 11× B-cell epitopes were assisted by KK linkers. Finally, 6× His were attached at the C-terminal region without a linker for the purification step. The final construct of the multi-epitope vaccine is represented in Figure 3.

### 3.6. Allergenicity and Antigenicity of the Multi-Epitope Vaccine

The similarity between the multi-epitope vaccine and human proteins was found in the identity for the 50S ribosomal protein L7/L12 but non-homology for other proteins by using BLAST. Then, AllerTOP was used to predict the allergenicity based on allergens and non-allergens. The results indicate that the vaccine constructs are likely to be non-allergenic. To evaluate the epitope antigenicity, the VaxiJen v.2.0 server was run with the threshold >0.5 for theparasite model. The server provided a score value of 0.6129, indicating the vaccine construct as a suitable antigen.

**Figure 4:**
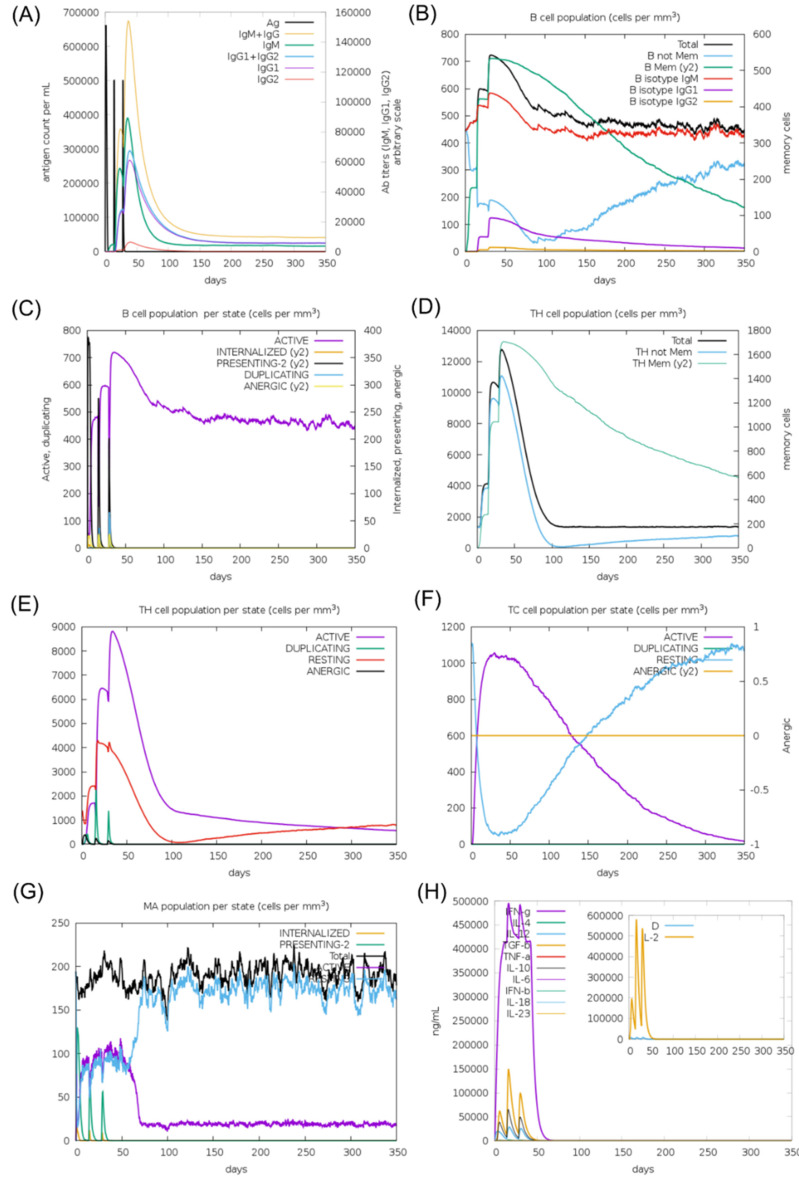
In silico simulation of the immune response for the multi-epitope vaccine. (**A**) Antigen and immunoglobulins, (**B**) B-cell population per state, (**C**) B-cell population, (**D**) T helper cell population per state, (**E**) T helper cell population, (**F**) T cytotoxic cell population per state, (**G**) macrophage population per state, and (**H**) production of cytokine and interleukins.

The in silico immune response results after the booster inoculation were significantly higher compared to primary inoculation. Increased titers of IgM, IgM + IgG, IgG1 + IgG2, and IgG1 antibodies were predicted, followed by a decrease in antigen concentration (Figure 4A). An increase in the active B-cell population was expected, particularly in memory B-cells (Figure 4B,C). T helper and T cytotoxic cells were detected with similar behavior to B-cells (Figure 4D,F). Among innate immune cells, increased macrophage activity was demonstrated (Figure 4G). In addition, higher levels of IFN-γ were expected (Figure 4H). These data support the potential of the CMEV to induce both humoral and cellular immune responses.

### 3.7. Physiochemical Characterization of Multi-Epitope Vaccine

The ProtParam tool was used to evaluate physicochemical parameters of the vaccine constructs based on the amino acid sequence. The molecular weight was approximately 66.7 kDa. The isoelectric point (pI) was 9.87, indicating that the protein is basic. There were 54 positively charged residues and 98 negatively charged residues. The instability index (II) was 38.77, suggesting that the protein is stable. The aliphatic index was 102.26, indicating that the higher the aliphatic index, the more stable the protein was over a wide temperature range. The estimated half-life of the vaccine construct was 30 h for mammalian reticulocytes, over 20 h for yeast, and over 10 h for *E. coli*. Furthermore, the SOLpro server, which predicts solubility when overexpressed, showed a good predictive value (0.8743), suggesting that the protein is soluble, consistent with the GRAVY results (Table 3).

### 3.8. Tertiary Structure Prediction of Multi-Epitope Vaccine

**Figure 5:**
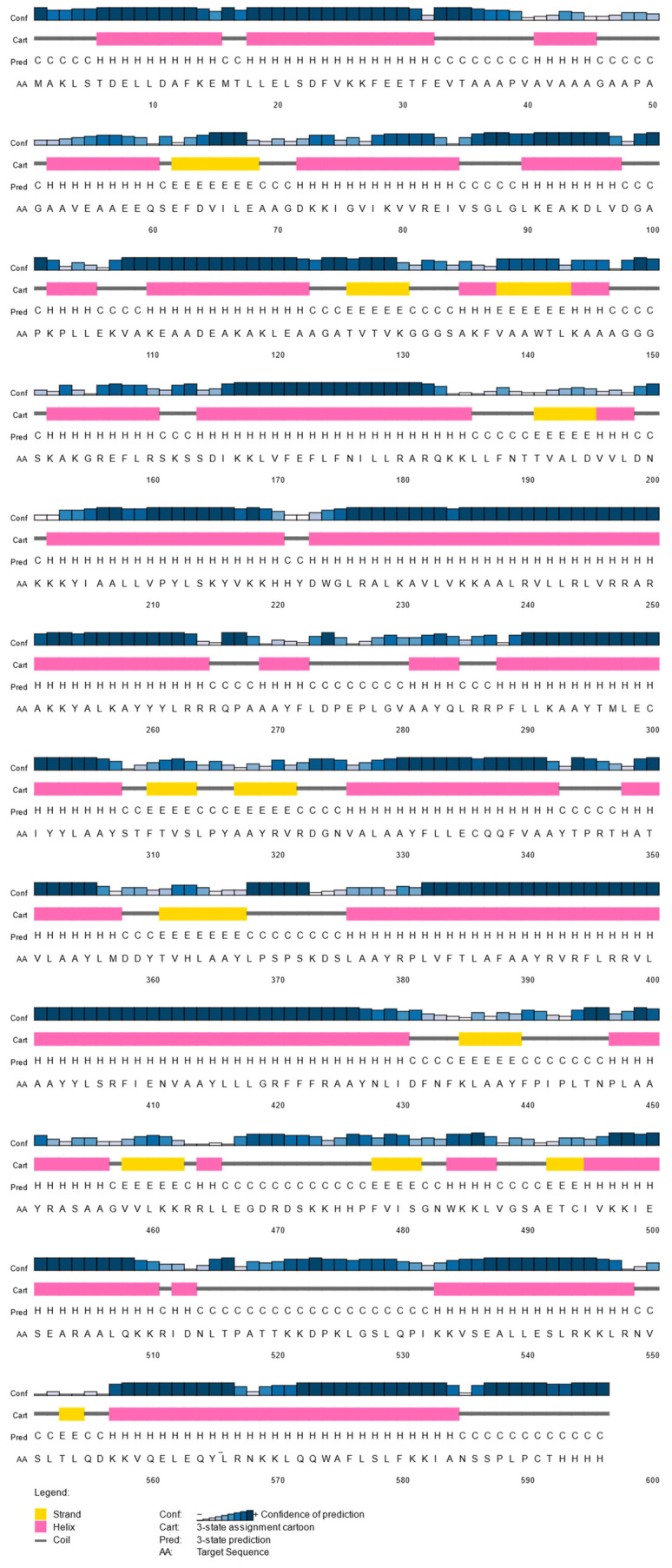
Predicted secondary structure of the designed multi-epitope vaccine construct showing alpha-helix, beta strands, and coils.

**Figure 6:**
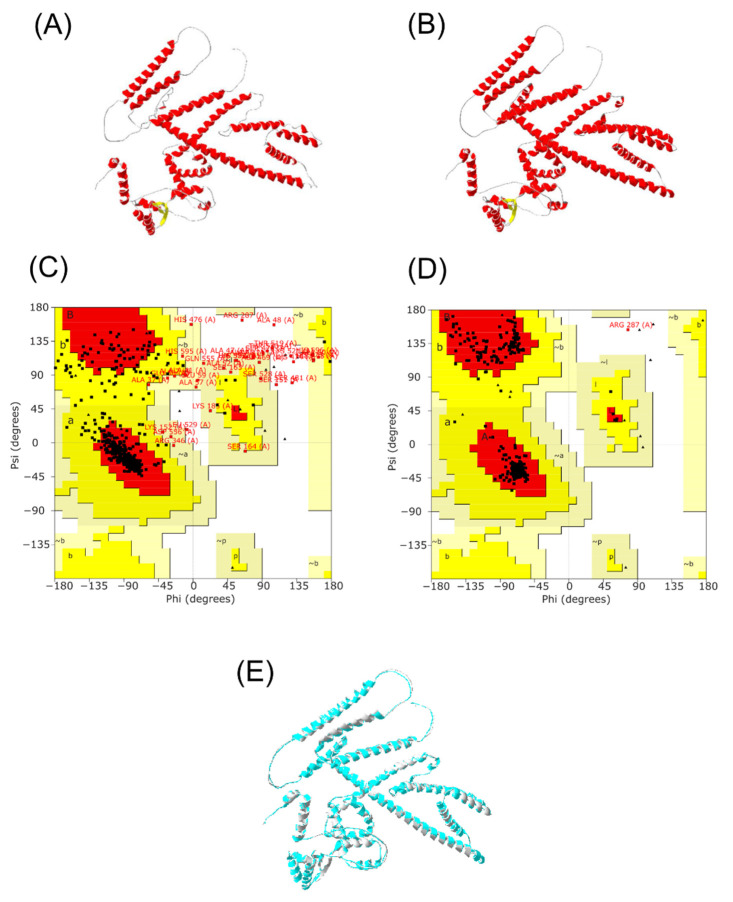
Multi-epitope vaccine modeling and refinement. (**A**) Tertiary structure generated by Alphafold2, (**B**) Refined tertiary structure obtained by GalaxyRefine server, (**C**) Ramachandran plot from initial model, (**D**) Ramachandran plot from refined model, (**E**) Merge structure predicted from Alphafold2 (gray) and refined structure from GalaxyRefine server (light blue).

Alphafold2 was run to construct the 3D structure of the final vaccine. Five potential models were obtained, then subsequently submitted to PROCHECK to study its stability, and the highest rank was collected. The secondary structure of the protein was composed of 63.9% α-helix, 3.3% β strand, and 32.8% coils (Figure 5). After choosing the best 3D model, a refinement was performed by the Galaxy Web server to improve structure quality. Five refined model were generated (Supplementary Table S4). The initial and refined structure were compared to select the best model based on the Ramachandran plot. The comparison demonstrated that the initial model showed 80.5% of the residues in the favorable region, 12.8% in the allowed regions, and 1.6% in the disallowed regions (Figure 6A,C). On the other hand, after the refinement, the model harbored 98.5%, 1.3%, and 0.2% of the residues in favored, allowed, and disallowed regions, respectively (Figure 6B,D). After refinement, the secondary structure information showed an increase in the α-helix region to 69.4%) (Figure 6E).

### 3.9. Prediction of Conformational Epitopes

**Figure 7:**
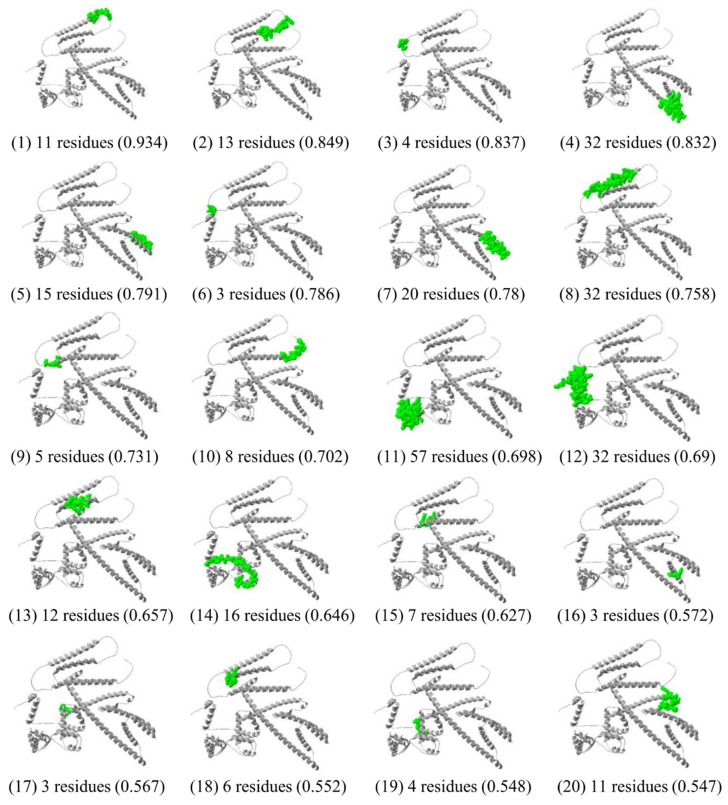
Conformational B-cell epitopes of the multi-epitope vaccine predicted by the ElliPro tool of IEDB analysis resource, represented in green surface.

From the Ellipro server, a total of 300 residues were distributed among 20 conformational B-cell epitopes, with scores ranging from 0.574 to 0.934 and sizes from 3 to 57 residues (Supplementary Table S5). Most B-cell epitopes were exposed on the surface of the multi-epitope vaccine (Figure 7).

### 3.10. Interaction of TLR4 and the Candidate Vaccine

**Figure 8:**
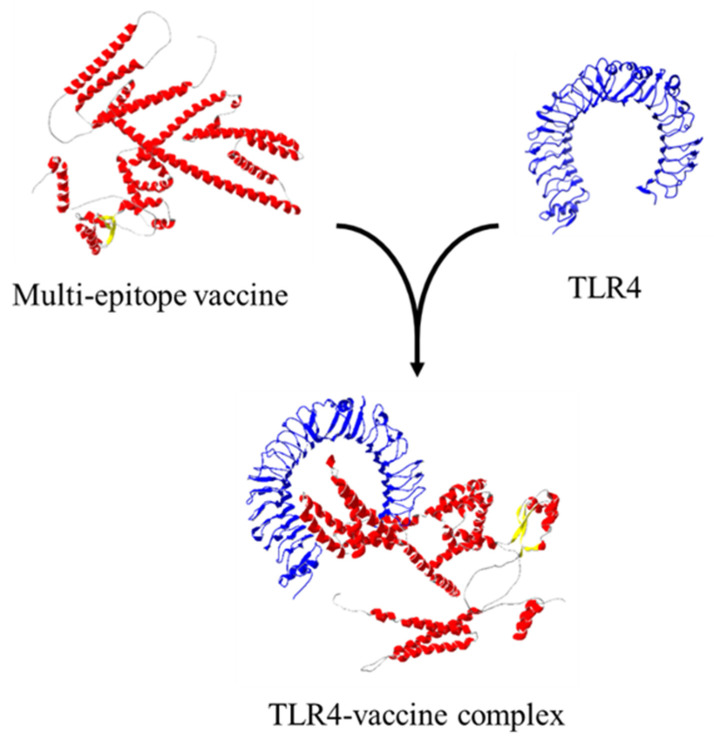
Ligand–protein interaction using ClusPro.

**Figure 9:**
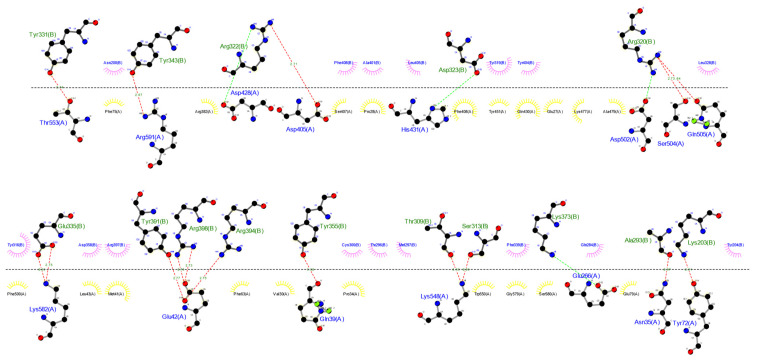
Interactions between ligand and receptor provided by the LIGPLOT v.2.2 software. Red lines represent hydrogen bonds, green lines represent a salt-bridge, yellow semi-circles denote hydrophobic interactions made by the ligand, and pink semi-circles represent hydrophobic interactions made by the receptor.

The ClusPro 2.0 server and LIGPLOT v2.2 software were run to simulate and analyze possible interactions between TLR4 (receptor) and ligand (multi-epitope vaccine), which were determined through the number of docked structures, the energy of the cluster center, and the lowest energy structure in the cluster based on the model score for a balanced coefficient set. The best model for the vaccine-receptor complex had 34 dock structures with the center energy approximately −1033.9 kJ.mol^−1^ and the lowest energy of −1119.0 kJ.mol^−1^ (Figure 8). This complex exhibited 16 hydrogen bonds, 4 salt bridges, and 16 hydrophobic interactions for the receptor and 20 for the ligand, which are involved in specificity and binding of the receptor and vaccine (Figure 9).

### 3.11. In Silico Cloning

**Figure 10:**
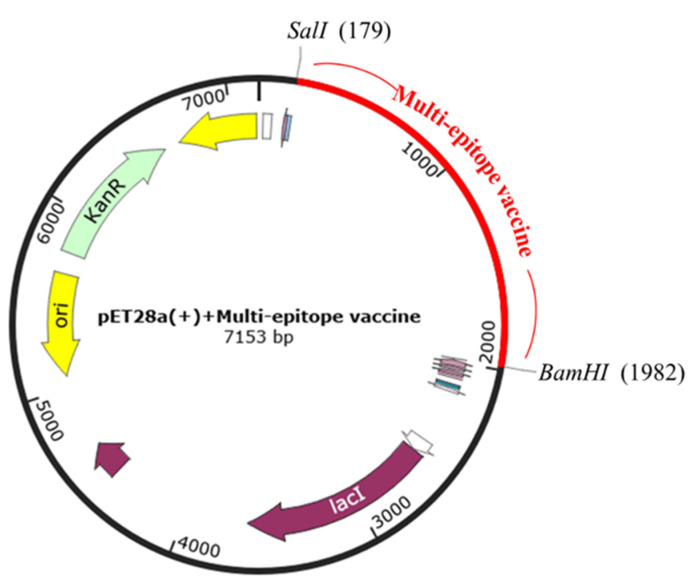
In silico cloning. Multi-epitope vaccine sequence was cloned into the pET28a(+) expression vector by *SalI* and *BamHI* restriction sites, represented by the red color.

To generate the vaccine-expression vector construct expressed in *E. coli*, the Jcat server was run to optimize the DNA sequence. The CAI value was 1.0, indicating the higher expression probability in *E. coli*. GC content reached 50.11%, which remains in the optimal range (30–70%). After that, the multi-epitope vaccine was inserted into the expression vector pET28a(+) with BamHI and SalI restriction enzyme site using SnapGene restriction enzyme cloning. Finally, expression vector pET28a(+) carrying the multi-epitope vaccine insert (7153 bp) is represented in Figure 10.

## 4. Discussion

In the present study, we designed a multi-epitope vaccine against both *L. martiniquensis* and *L. orientalis* which are one of the causative agents of the parasitic disease, Leishmaniasis. This is the first report of the exploration of potential antigenic epitopes of proteins that are conserved among two *Leishmania* parasites, which are recently emerging in Thailand. Multi-epitope vaccines have been designed against a single species of other *Leishmania* parasites in previous studies [22,36,49,50], and the current study would also expand those efforts.

The strategy of this study is based on reverse vaccinology [51] and immuno-informatics [52,53]. The genomes of *L. orientalis* isolate PCM2 and *L. martiniquensis* isolate PCM3 yielded 21 and 30 putative antigenic proteins, respectively. Sixteen proteins were identified as putative antigenic proteins common between these two isolates, and a vaccine was designed based on these proteins. Recently, highly accurate genomes for *L. martiniquensis* and *L*. *orientalis,* isolated in northern Thailand, were reported [54,55]. In this study, however, candidate antigens were selected based on the data from draft genomes of the two species isolated in southern Thailand [25], and more candidates may be found if highly accurate sequence information becomes available. It should be noted that the results of this study are based on the information available at this time, and further studies on *L. martiniquensis* and *L. orientalis* isolates from diverse regions are needed.

For the construction of a multi-epitope vaccine, rational strategies based on antigenicity, physicochemical and structural properties, and interaction with immune receptors were used. We combined the candidate epitopes together with specific linkers such as GGGS, KK, and AAY. The main roles of these linkers are structural flexibility and rigidity, and antigen processing and presentation [31]. Additionally, we added a protein (50S ribosomal protein L7/L12) working as an adjuvant at the N-terminal to enhance the immunogenicity of this vaccine. This is based on a previous study that showed that the 50S ribosomal protein L7/L12 could induce the maturation of dendritic cells, which activate naïve T-cells, resulting in the polarization of CD4+ and CD8+ T cells to secrete IFN-γ [56]. In addition, PADRE peptides, which are a universal T helper epitope that improve antibody immune responses caused by the recombinant vaccine against malaria [48], are applied to the vaccine. Indeed, the results of the in silico immune response simulation of this study predicted the secretion of high levels of IFN-γ and a long-lasting cellular response. These data indicate that our candidate multi-epitope vaccine would induce an effective immune response and protect the host against the disease.

The primary immune response that occurs during *Leishmania* infection is thought to be through cellular immunity. After infection, interleukin-12 (IL-12) is expressed by antigen-presenting cells (APCs) and promotes the differentiation of antigen-specific CD4+ T cells into IFN-γ and TNF-producing Th1 cells, which activate macrophages to kill internalized parasites [57,58]. In addition, IL-12 also activates CD8+ T cells that promote the expression of type I cytokines such as TNF or IFN-γ and cytotoxic molecules such as granzymes and perforin, leading them to kill infected cells [59,60]. Therefore, *Leishmania* vaccine candidates are required to have the ability to induce Th1 rather than Th2-type responses [13] in immune cells. The results of the ligand and TLR4 binding capacity analysis performed in this study suggest that a Th1-type T-cell immune response can be elicited by the designed vaccine, resulting in efficacy against the parasites.

The physicochemical characteristics of the designed multi-epitope vaccine suggested that the protein would be expressed in bacterial cells. The predicted half-life in *E. coli* (10 h) and the stability of the molecule (instability index 38.77, aliphatic index 102.27, and solubility 0.8743) suggested that *E. coli* could be used as a platform for heterologous expression. Therefore, we optimized the codon usage of the vaccine candidates based on the *E. coli* K12 strain and performed in silico cloning in a common expression vector, pET28a(+).

## 5. Conclusions

In this study, the CMEV against both *L. martiniquensis* and *L. orientalis*, which cause Leishmaniasis in Thailand, were designed. Immunogenic, physicochemical, and structural properties suggest that our vaccine candidate is expected to be appropriately expressed in the bacterial expression system and has the potential to induce immune responses in the host. This is the first report of a vaccine candidate that is expected to be effective against these two species. However, further in vitro and in vivo studies are needed to validate the efficacy of this novel candidate CMEV. The present study proposes the experimental validation of the efficacy of the candidate vaccine construct against the two *Leishmania*.

## Figures and Tables

**Table 1:** Identity of conserved antigenic proteins from *L. martiniquensis* and *L. orientalis*.

	*L. Martiniquensis*	*L. Orientalis*	Distance Value	% Identity
1	CN030422.1.g3678	k59_6959.g7826	0.114354	79.57
2	CN030417.1.g2464	k59_8122.g3660	0.109277	84.94
3	CN030426.1.g5328	k59_3810.g2871	0.0946353	88.52
4	CN030405.1.g85	k59_8079.g2401	0.14275	80.25
5	CN030411.1.g1102	k59_7325.g3419	0.109568	79.16
6	CN030414.1.g1733	k59_1681.g2029	0.279142	69.95
7	CN030430.1.g7697	k59_6133.g7748	0.061051	88.03
8	CN030412.1.g1340	k59_4380.g3725	0.242199	74.82
9	CN030421.1.g3586	k59_9180.g3154	0.15986	81.75
10	CN030429.1.g7028	k59_748.g6249	0.0319805	94.73
11	CN030408.1.g620	k59_7892.g4284	0.250543	84.01
12	CN030431.1.g7874	k59_2726.g2266	0.184671	68.98
13	CN030402.1.g9136	k59_5929.g1357	0.100062	81.01
14	CN030402.1.g9114	k59_9933.g1615	0.15858	80.45
15	CN030425.1.g4940	k59_5159.g3601	0.11762	81.53
16	CN030420.1.g3164	k59_328.g2467	0.288368	63.49

**Table 2:** Selected B-cell epitopes, CTL epitopes, and HTL epitopes.

No.	*L. martiniquensis*	*L. orientalis*	B-Cell	CTL	HTL
1	CN030422.1.g3678	k59_6959.g7826	RRLLEGDRDS	FLDPEPLGV	KAKGREFLRSKSSDI
2	CN030417.1.g2464	k59_8122.g3660		QLRRPFLLK	
3	CN030426.1.g5328	k59_3810.g2871	HHPFVISGNW	TMLECIYYL	LVFEFLFNILLRARQ
4	CN030405.1.g85	k59_8079.g2401	LVGSAETCIV	STFTVSLPY	LLFNTTVALDVVLDN
5	CN030411.1.g1102	k59_7325.g3419		RVRDGNVAL	KYIAALLVPYLSKYV
6	CN030414.1.g1733	k59_1681.g2029	IESEARAALQ	FLLECQQFV	
7	CN030430.1.g7697	k59_6133.g7748		TPRTHATVL	
8	CN030412.1.g1340	k59_4380.g3725		LMDDYTVHL	
9	CN030421.1.g3586	k59_9180.g3154	RIDNLTPATT	LPSPSKDSL	
10	CN030429.1.g7028	k59_748.g6249	DPKLGSLQPI	RPLVFTLAF	HHYDWGLRALKAVLV
11	CN030408.1.g620	k59_7892.g4284	VSEALLESLR	RVRFLRRVL	AALRVLLRLVRRARA
12	CN030431.1.g7874	k59_2726.g2266	LRNVSLTLQD	YLSRFIENV	
13	CN030402.1.g9136	k59_5929.g1357	VQELEQYLRN	LLLGRFFFR	YALKAYYYLRRRQPA
14	CN030402.1.g9114	k59_9933.g1615	LQQWAFLSLF	NLIDFNFKL	
15	CN030425.1.g4940	k59_5159.g3601	IANSSPLPCT	FPIPLTNPL	
16	CN030420.1.g3164	k59_328.g2467		RASAAGVVL	

**Table 3:** Physiochemical properties of the multi-epitope vaccine and the HTL, CTL, and B-cell epitopes.

Protein/ Peptide	Number of Amino Acids	Molecular Weight (kDa)	Isoelectric Point	Instability Index	Aliphatic Index	GRAVY *
HTL	117	13.8	10.91	58.99	116.75	−0.196
CTL	189	21.3	9.39	33.15	107.09	0.514
B-cell	130	14.8	10.16	51.26	94.57	−0.668
Multi-epitope vaccine	598	66.7	9.87	38.77	102.27	−0.010

GRAVY *: Grand average of hydropathicity. The multi-epitope vaccine was constructed using amino acid sequences of adjuvants (50S ribosomal protein L7/L12 and PADRE peptide) followed by selected HTL, CTL, and B-cell epitopes from antigenic proteins and 6xHis connected by a suitable linker.

## Data Availability

The datasets generated and analyzed during the current study are available from the corresponding author on reasonable request.

## Supplementary Materials

The following are available online at https://www.mdpi.com/article/10.3390/biology11101460/s1, Table S1: Details of B-cell epitopes., Table S2: Predicted A2, A3 and B7 supertype CTL epitopes., Table S3: Details of HTL epitopes., Table S4: Model after refinement using Galaxy Web., Table S5: Conformational B-Cell epitopes from refined model.
