# Supplementary material for: Design of a Chimeric Multi-Epitope Vaccine (CMEV) against Both Leishmania martiniquensis and Leishmania orientalis Parasites Using Immunoinformatic Approaches
Source: Biology (Basel). 2022 Oct 5;11(10):1460. doi: 10.3390/biology11101460 (PMC9598663; doi:10.3390/biology11101460)
Supplement: Supplementary file 1 [file biology-11-01460-s001.zip › biology-1924550-supplementary11.pdf]

Supplementary data

Supplementary Table S1. Details of B-cell epitopes.

	L. martiniquensis	*L. orientalis*	**B-CELL EPITOPE**	**Antigenicity**
1	CN030422.1.g3678	k59_6959.g7826	RRLLEGDRDS	1.1104
2	CN030417.1.g2464	k59_8122.g3660	REWRLVHRLL	-0.3635
3	CN030426.1.g5328	k59_3810.g2871	HHPFVISGNW	1.591
4	CN030405.1.g85	k59_8079.g2401	LVGSAETCIV	0.6586
5	CN030411.1.g1102	k59_7325.g3419	ELLETVMDMV	-0.0358
6	CN030414.1.g1733	k59_1681.g2029	IESEARAALQ	1.1867
7	CN030430.1.g7697	k59_6133.g7748	RMRANQVYSI	0.4402
8	CN030412.1.g1340	k59_4380.g3725	THVNALREVV	0.0907
9	CN030421.1.g3586	k59_9180.g3154	RIDNLTPATT	0.6515
10	CN030429.1.g7028	k59_748.g6249	DPKLGSLQPI	0.5804
11	CN030408.1.g620	k59_7892.g4284	VSEALLESLR	0.6877
12	CN030431.1.g7874	k59_2726.g2266	LRNVSLTLQD	0.6631
13	CN030402.1.g9136	k59_5929.g1357	VQELEQYLRN	0.7325
14	CN030402.1.g9114	k59_9933.g1615	LQQWAFLSLF	0.673
15	CN030425.1.g4940	k59_5159.g3601	IANSSPLPCT	1.0008
16	CN030420.1.g3164	k59_328.g2467	LPSPQDIEHA	0.1363

Supplementary Table S2. Predicted A2, A3 and B7 supertype CTL epitopes.

	CTL epitope								
	Supertype A2			Supertype A3			Supertype B7		
	Amino acid sequence	Score	Antigenicity	Amino acid sequence	Score	Antigenicity	Amino acid sequence	Score	Antigenicity
1	FLDPEPLGV	1.4314	1.2248	KTLAAAMHK	1.5889	0.4143	IPANSTCLL	1.6319	0.4149
2	FLFDESNIL	1.4269	-0.2443	QLRRPFLLK	1.6515	0.6344	LPRSVRIIF	1.7595	0.4836
3	TMLECIYYL	1.5528	0.9859	RLLPHVLAK	1.5868	-0.1514	APSWCSTVL	1.8113	0.5628
4	ALLSFPSSV	1.3667	0.2795	STFTVSLPY	1.446	0.9065	NPVSYTVSF	1.6911	0.2925
5	SMLARDFGV	1.3756	-0.3371	LLYLPFCYK	1.6866	0.8986	RVRDGNVAL	1.8336	0,9381
6	FLLECQQFV	1.4063	1.509	LLLSSSVSY	1.2849	0.219	IPLSARHPL	1.7472	0.5824
7	FLTQDVLAV	1.4475	0.3596	RLHQPSLEK	1.7119	-0.2921	TPRTHATVL	1.7781	0.917
8	LMDDYTVHL	1.502	0.6336	RQHHIAFSY	1.5722	0.008	APRSASEAL	1.7835	0.0269
9	FMTHNIIQL	1.4006	0.328	CVTSPILSK	1.4371	0.4913	LPSPSKDSL	1.6597	0.5384
10	TLYDYYFDL	1.5407	0.997	RTLAPQLKK	1.637	0.1005	RPLVFTLAF	1.82	1.403
11	SLAAALAAV	1.3402	0.1461	RSYTMPSLY	1.6739	0.2513	RVRFLRRVL	1.8026	2.0869
12	YLSRFIENV	1.4426	1.6355	RSFLWPLYR	1.4839	0.7472	SPSEFRAAL	1.8343	0.8273
13	AVMAATWSV	1.4432	0.5309	LLLGRFFFR	1.5952	2.3608	WPVPMRGIF	1.6386	0.4248
14	NLIDFNFKL	1.4685	1.0674	TTLFVSLVK	1.4836	0.7296	SPQPATAAL	1.7869	0.6939
15	KLFDFGPLL	1.5438	0.4642	KLHFSIALR	1.638	0.5372	FPIPLTNPL	1.6645	1.1079
16	YMWSTDVCL	1.4589	1.0686	RLAWAALQR	1.5326	-0.8802	RASAAGVVL	1.5219	1.0727

Supplementary Table S3. Details of HTL epitopes.

	L. martiniquensis	*L. orientalis*	HTL epitope				
			**Allele**	**Amino acid sequence**	**IC50**	**SMM align IC50 (nM)**	**Antigenicity**
1	CN030422.1.g3678	k59_6959.g7826	HLA-DRB1*07:01	KAKGREFLRSKSSDI	4	0.128	0.6689
2	CN030417.1.g2464	k59_8122.g3660	HLA-DRB1*01:01	HYDFGLRALKSVLVA	2	0.064	0.3061
3	CN030426.1.g5328	k59_3810.g2871	HLA-DRB1*11:01	LVFEFLFNILLRARQ	5	0.148	1.2509
4	CN030405.1.g85	k59_8079.g2401	HLA-DRB1*07:01	LLFNTTVALDVVLDN	6	0.165	0.5229
5	CN030411.1.g1102	k59_7325.g3419	HLA-DRB1*15:01	KYIAALLVPYLSKYV	6	0.165	0.9843
6	CN030414.1.g1733	k59_1681.g2029	HLA-DRB4*01:01	SQATLVLERLQRLLW	13	0.237	0.4712
7	CN030430.1.g7697	k59_6133.g7748	HLA-DRB1*04:01	QPQFLRLLSFTALRT	19	0.272	0.2185
8	CN030412.1.g1340	k59_4380.g3725	HLA-DRB1*01:01	AEYVSGLLMLRAVLA	2	0.064	0.4838
9	CN030421.1.g3586	k59_9180.g3154	HLA-DRB1*01:01	SDPVPFYTLKPVAVR	2	0.064	0.1528
10	CN030429.1.g7028	k59_748.g6249	HLA-DRB1*01:01	HHYDWGLRALKAVLV	2	0.064	0.5008
11	CN030408.1.g620	k59_7892.g4284	HLA-DRB1*11:01	AALRVLLRLVRRARA	5	0.148	0.5194
12	CN030431.1.g7874	k59_2726.g2266	HLA-DRB1*04:01	FGFFRGVSATSEFYA	29	0.3	-0.4509
13	CN030402.1.g9136	k59_5929.g1357	HLA-DRB1*11:01	YALKAYYYLRRRQPA	3	0.1	0.5762
14	CN030402.1.g9114	k59_9933.g1615	HLA-DRB1*15:01	TEDQLLRFLKGFYSV	14	0.243	-0.405
15	CN030425.1.g4940	k59_5159.g3601	HLA-DRB1*07:01	AAFLSTAASMVGRRN	2	0.064	0.1927
16	CN030420.1.g3164	k59_328.g2467		NF			

Supplementary Table S4. Model after refinement using Galaxy Web

**Model**	**GDT-HA**	**RMSD**	**MolProbity**	**Clash score**	**Poor rotamers**	Rama favored
**Initial**	**1**	**0**	**3.154**	**15.3**	**9.8**	81.7
MODEL 1	0.898	0.565	0.858	1.3	0	99.3
MODEL 2	0.9139	0.552	0.876	1.4	0.2	99
MODEL 3	0.8972	0.59	0.958	1.9	0.2	98.8
MODEL 4	0.9202	0.537	0.972	2	0	98.3
MODEL 5	0.9059	0.531	0.893	1.5	0.2	98.5

Supplementary Table S5. Conformational B-Cell epitopes from refined model.

Residues	Number of residues	Score
A:R511, A:I512, A:D513, A:N514, A:L515, A:T516, A:P517, A:A518, A:T519, A:T520, A:K521	11	0.934
A:K522, A:D523, A:P524, A:K525, A:L526, A:G527, A:S528, A:L529, A:Q530, A:P531, A:I532, A:K533, A:K534	13	0.849
A:H475, A:H476, A:P477, A:F478	4	0.837
A:T361, A:V362, A:H363, A:L364, A:A365, A:A366, A:Y367, A:L368, A:P369, A:S370, A:P371, A:S372, A:K373, A:D374, A:S375, A:L376, A:A377, A:A378, A:Y379, A:R380, A:P381, A:L382, A:V383, A:F384, A:T385, A:L386, A:A387, A:F388, A:A389, A:A390, A:Y391, A:R392	32	0.832
A:S308, A:T309, A:F310, A:T311, A:V312, A:S313, A:L314, A:P315, A:Y316, A:A317, A:A318, A:Y319, A:R320, A:V321, A:R322	15	0.791
A:D471, A:S472, A:K473	3	0.786
A:D323, A:G324, A:N325, A:V326, A:A327, A:L328, A:A329, A:A330, A:Y331, A:F332, A:L333, A:L334, A:E335, A:C336, A:Q337, A:Q338, A:F339, A:A341, A:A342, A:T344	20	0.78
A:V479, A:I480, A:S481, A:G482, A:N483, A:W484, A:K485, A:K486, A:L487, A:V488, A:G489, A:S490, A:A491, A:E492, A:T493, A:C494, A:I495, A:V496, A:K497, A:K498, A:I499, A:E500, A:S501, A:E502, A:A503, A:R504, A:A505, A:A506, A:L507, A:Q508, A:K509, A:K510	32	0.758
A:L465, A:L466, A:E467, A:G468, A:D469	5	0.731
A:S587, A:P588, A:L589, A:P590, A:C591, A:T592, A:H593, A:H594	8	0.702
A:S61, A:L67, A:E68, A:A69, A:A70, A:G71, A:D72, A:K73, A:K74, A:I75, A:G76, A:V77, A:I78, A:K79, A:V80, A:V81, A:R82, A:E83, A:I84, A:V85, A:S86, A:G87, A:L88, A:G89, A:L90, A:K91, A:E92, A:A93, A:K94, A:D95, A:L96, A:V97, A:D98, A:G99, A:A100, A:P101, A:K102, A:V108, A:A109, A:K110, A:E111, A:A112, A:A113, A:D114, A:E115, A:A116, A:K117, A:A118, A:K119, A:L120, A:E121, A:A122, A:A123, A:G124, A:A125, A:T126, A:V127	57	0.698
A:M1, A:A2, A:K3, A:L4, A:S5, A:E8, A:L9, A:D11, A:A12, A:F13, A:K14, A:E15, A:M16, A:T17, A:L18, A:L19, A:E20, A:L21, A:S22, A:D23, A:F24, A:V25, A:K26, A:K27, A:F28, A:E29, A:E30, A:T31, A:F32, A:V34, A:T35, A:A36	32	0.69
A:V535, A:S536, A:E537, A:A538, A:L539, A:L540, A:E541, A:S542, A:L543, A:R544, A:K545, A:K546	12	0.657
A:A38, A:P39, A:V40, A:A41, A:V42, A:A43, A:A44, A:A45, A:G46, A:A47, A:A48, A:P49, A:A50, A:G51, A:A52, A:A53, A:V54, A:E55	16	0.646
A:S454, A:A455, A:G457, A:V458, A:V459, A:K461, A:K462	7	0.627
A:M357, A:D359, A:Y360	3	0.572
A:D223, A:W224, A:R227	3	0.567
A:N549, A:V550, A:S551, A:L552, A:T553, A:L554	6	0.552
A:K215, A:K219, A:H220, A:Y222	4	0.548
A:R263, A:Q266, A:P267, A:A268, A:A269, A:A270, A:Y271, A:L273, A:D274, A:P275, A:E276	11	0.547
